# Anaesthesia management of a giant retroperitoneal liposarcoma: a case report and literature review

**DOI:** 10.3389/fsurg.2026.1901393

**Published:** 2026-10-08

**Authors:** Fei Huo, Bin An, Hansheng Liang, Liang Sun, Mengmeng Xiao, Chenghua Luo, Yi Feng

**Affiliations:** 1 Department of Anesthesiology, Peking University People ’s Hospital, Beijing, China; 2 Department of Retroperitoneal Oncology, National Center for Trauma Medicine, Peking University People's Hospital, Beijing, China

**Keywords:** anaesthesia management, giant, multidisciplinary team, retroperitoneal liposarcoma, tumor compression

## Abstract

Soft tissue sarcomas account for less than 1% of all malignant tumors, of which 10∼15% are located in the retroperitoneum, and liposarcoma constitutes 20% of all soft tissue sarcomas. Of note, retroperitoneal liposarcoma is a rare malignant tumor, with recurrence and asymptomatic characteristics at the initial stage until the liposarcoma is large enough to compress abdominal organs. Those larger than 20 kg, known as "giant liposarcomas," are extremely rare. Most retroperitoneal liposarcoma represents expansive growth, and lymph node metastasis or distant metastasis is rarely reported, making surgical resection as the most effective treatment. Because giant retroperitoneal liposarcomas can cause substantial compression of abdominal and retroperitoneal structures, perioperative management may be complicated by impaired venous return, respiratory compromise, hemodynamic instability, difficult tumor dissection, and major blood loss. Current consensus emphasizes management of primary retroperitoneal sarcoma in experienced multidisciplinary centers. In addition, recent perioperative literature highlights the importance of individualized preoperative optimization, invasive hemodynamic monitoring, goal-directed fluid therapy, adequate blood-product preparation, and structured postoperative care. In this context, we reported a case of a giant retroperitoneal liposarcoma with a diameter 52 cm× 38 cm×30 cm and a weight of 31 kg. Through multidisciplinary team (MDT) consultation, the tumor compressing the surrounding organs was successfully removed.

## Key clinical message

Management of giant retroperitoneal liposarcoma requires a multidisciplinary team approach, where the anaesthesiologist plays a pivotal role in optimizing perioperative care and ensuring patient safety during complex tumor resections.

## Introduction

Soft tissue sarcomas account for less than 1% of all malignant tumors, of which 10～15% are located in the retroperitoneum, and liposarcoma constitutes 20% of all soft tissue sarcomas (1). Of note, retroperitoneal liposarcoma is a rare malignant tumor, with recurrence and asymptomatic characteristics at the initial stage until the liposarcoma is large enough to compress abdominal organs. Those larger than 20 kg, known as “giant liposarcomas,” are extremely rare (2). Most retroperitoneal liposarcoma represents expansive growth, and lymph node metastasis or distant metastasis is rarely reported, making surgical resection as the most effective treatment (3). Because giant retroperitoneal liposarcomas can cause substantial compression of abdominal and retroperitoneal structures, perioperative management may be complicated by impaired venous return, respiratory compromise, hemodynamic instability, difficult tumor dissection, and major blood loss. Current consensus emphasizes management of primary retroperitoneal sarcoma in experienced multidisciplinary centers (11). In addition, recent perioperative literature highlights the importance of individualized preoperative optimization, invasive hemodynamic monitoring, goal-directed fluid therapy, adequate blood-product preparation, and structured postoperative care. In this context, we reported a case of a giant retroperitoneal liposarcoma with a diameter 52 cm × 38 cm × 30 cm and a weight of 31 kg. Through multidisciplinary team (MDT) consultation, the tumor compressing the surrounding organs was successfully removed.

## Case report

A 38-year-old male patient weighing 92 kg with a BMI of 31.1 kg/m^2^ was scheduled for reoperation due to the recurrence of retroperitoneal liposarcoma 6 years after surgery. No relevant psychosocial history was identified. The abdomen was distended, and large masses were palpable up to the xiphoid process, down to the symphysis pubis, and lateral to the midaxillary lines, with toughness and poor flexibility. Laboratory tests showed no obvious abnormalities. Enhanced CT indicated that the colon and small intestine surrounded the tumor, and the abdominal aorta, inferior vena cava, kidney and ureter were closely attached to the tumor. Almost all organs in the abdominal cavity were compressed and hydronephrosis developed in the right kidney (Figures 1A,B). The preoperative MDT convened by the anesthesiologists included retroperitoneal oncologic surgeons, urologists, gastrointestinal surgeons, vascular surgeons and intensivists to discuss perioperative tumor resection-related topics (Figure 2), and subsequent key contents of MDT consensus were reached (Table 1).

**Figure 1:**
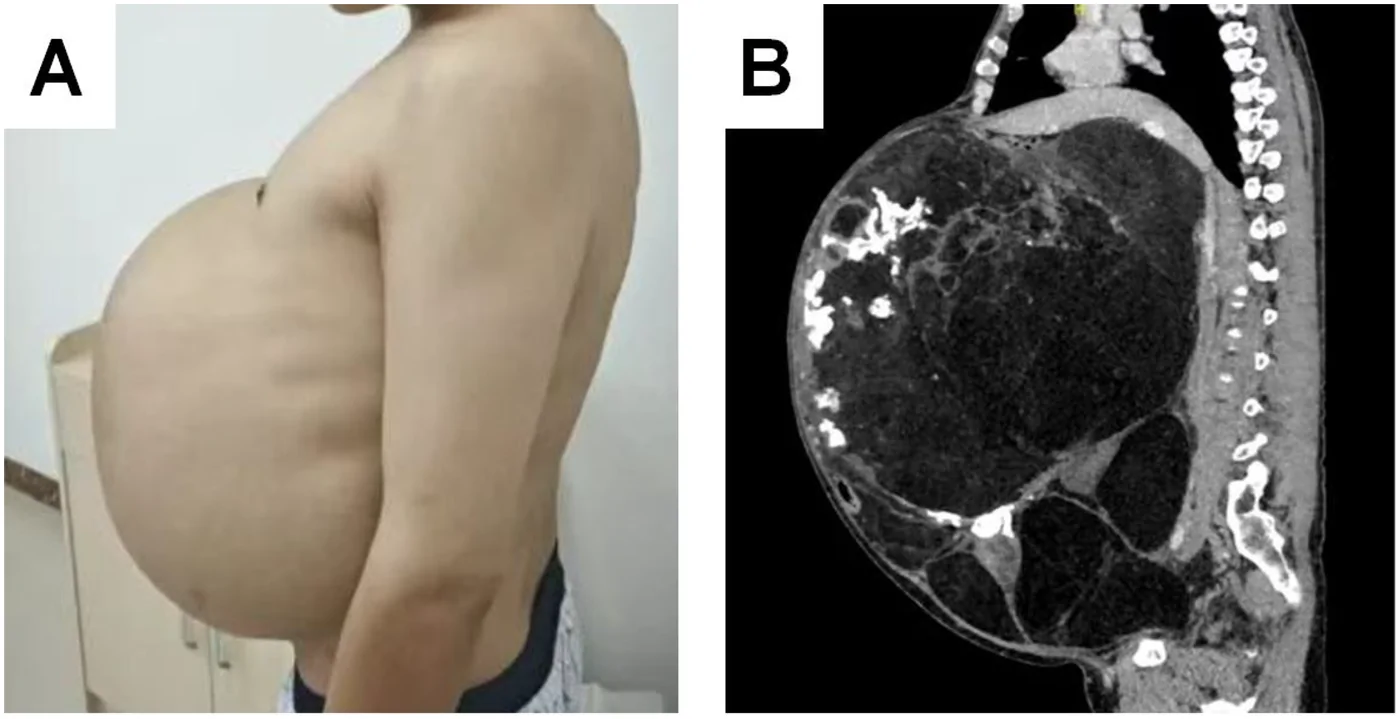
The overall view of the tumor **(A)**, preoperative image**; (B)**, preoperative computed tomography scan.(saggital plane).

**Figure 2:**
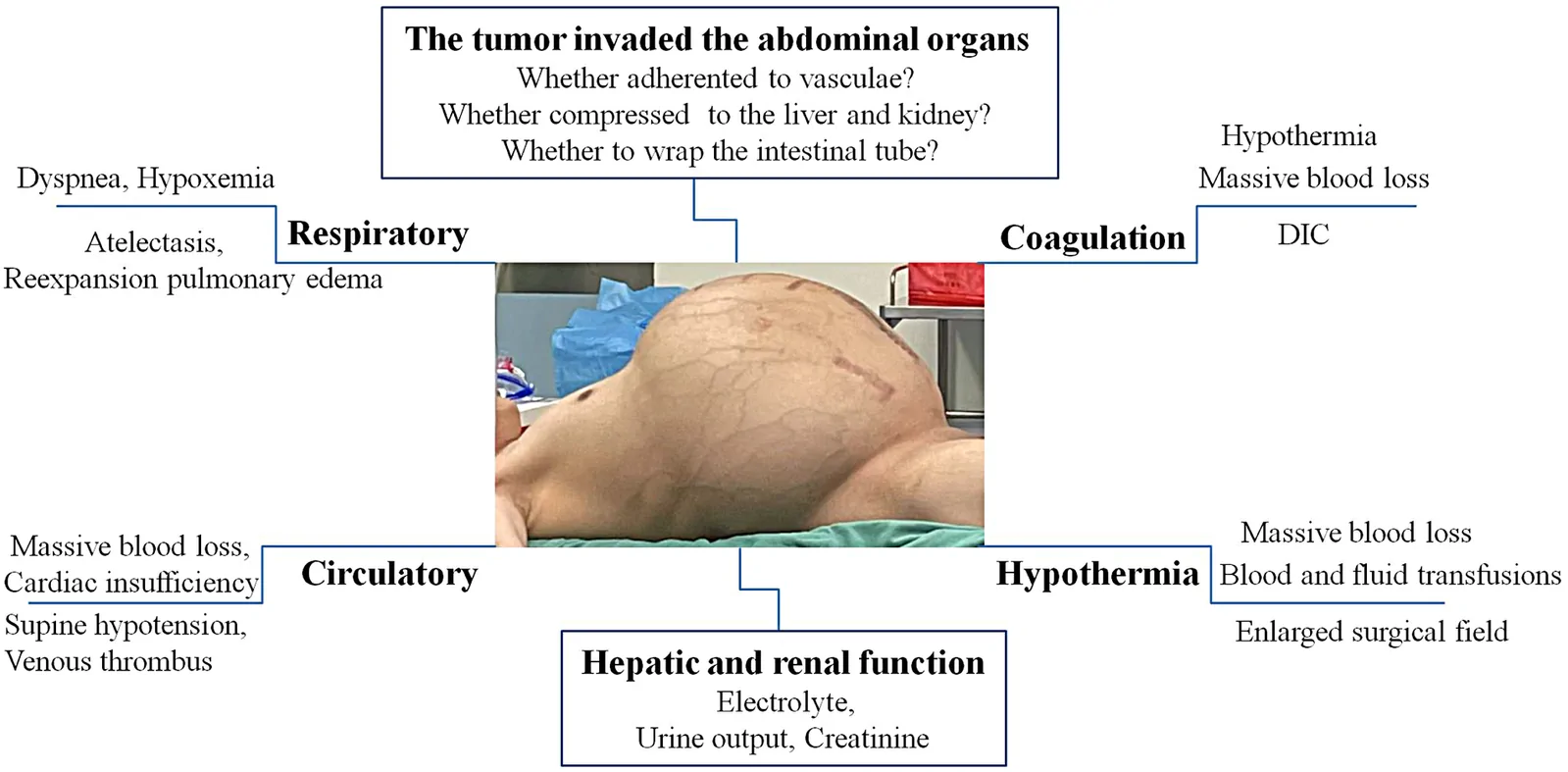
Issues to be considered in preoperative multidisciplinary consultation for patients with giant abdominal liposarcoma.

**Table 1:** Key contents of MDT consensus.

Attention Category	Specific Considerations
Surgical attention	1) To prevent accidental injury of the right ureter compressed by liposarcoma during operation
	2) Liposarcoma wrapped the colon and adhesions should be released thoroughly, and partial colectomy should be performed if necessary
	3) The separation of the adhesions between the inferior vena cava, iliac artery, and renal vessels and the tumor.
Anaesthesia attention	1) Considerations: massive bleeding may occur when separating tumor from blood vessels. Regimens: two peripheral venous access of the upper extremities with large diameter needle (18G) and central venous access before operation; RBCS, plasma, platelets, and fibrinogen and prothrombin complexes were stocked
	2) The challenges of cardiopulmonary and coagulation abnormalities such as supine hypotension, hypoxia, hypothermia, electrolyte disturbances, and reexpansion pulmonary edema.
ICU attention	1) Adequate anti-inflammatory measures, analgesia, and blood product supplementation

RBCS, red blood cells.

Restrictive fluid infusion, reverse Trendelenburg position, and manual left tumor displacement were performed before anaesthesia induction. Vital signs after admission showed as follows: Bp 119/84 mmHg, HR 76 bpm, SpO_2_ 98%, RR 22 bpm. After securing large-bore venous access, an arterial catheter was placed under local anaesthesia before anaesthesia induction. Anaesthesia was smoothly induced with 2 mg midazolam, 16 mg etomidate, and 12 mg cisatracurium. Tracheal intubation was completed, and mechanical ventilation was initiated using a tidal volume-controlled mode with a positive end-expiratory pressure (PEEP) of 5 cm H_2_O. The tidal volume was set at 400 mL, maintaining an end-tidal CO_2_ (EtCO_2_) between 35 and 45 mmHg. A central venous catheter (CVC) was then inserted in the right internal jugular vein. Anaesthesia was maintained with propofol (10 mg/mL) at 20–30 mL/h, remifentanil (40 µg/mL) at 10–15 mL/h, and sevoflurane at 1%, supplemented by intermittent cisatracurium boluses. Dopamine was infused at 5 µg/kg/min prior to the tumor resection. The patient was positioned in lithotomy position, and a right ureteral stent and urinary catheter were placed. Surgery proceeded with sequential dissection of the left abdominal wall, right abdominal wall, retroperitoneal space, right posterolateral wall, and finally, the left posterolateral wall. Once after the tumor resection (tumor size: 52 cm × 38 cm × 30 cm), norepinephrine was initiated at 0.05 µg/kg/min. Intraoperative fluid management included 2000mL of lactated Ringer's solution, 1,000 mL of hydroxyethyl starch (HES), 100 mL of 20% albumin, 2 units of packed red blood cells, and 400 mL of plasma, with a total input volume of 3,500 mL. Estimated blood loss was 600 mL, and urine output was 750 mL, with a total output of 1,350 mL. Perioperative blood gas tests were described in Table 2.

**Table 2:** Perioperative blood gas tests.

Parameter	Preoperatively	Before the resection of tumor	After the resection of tumor
PH	7.400	7.303	7.338
PaCO_2_	38.6	47.5	42.7
PaO_2_	139	175	195
Na+	140	141	141
K+	3.7	3.9	3.9
Ca++	1.16	1.12	1.08
Glu	4.2	7.8	8.7
Lac	0.9	0.9	1.2
Hct	45.5	40.3	34.7
HCO3-	23.9	21.9	22.1
BE-b	−2.2	−3.1	−2.8
SO_2_%	100	100	100
Hb	14.8	13.1	11.3
FiO_2_%	45	70	70

Glu, glucose; Lac, lactic acid; Hct, hematocrit; BE, base excess; FiO_2_%, fraction of inspired oxygen.

Postoperatively, the patient was transferred to the intensive care unit (ICU). His vital signs were stable, with a blood pressure of 111/73 mmHg, heart rate of 63 bpm, and oxygen saturation (SpO_2_) of 100% after admission. Postoperative bundled care included ventilatory support, continuous cardiac monitoring, anti-inflammatory therapy, and analgesia. The patient was successfully extubated 5 h after surgery, and experienced no postoperative respiratory or urinary complications, The patient was discharged from the ICU on postoperative day 3. The total hospital stay was 15 days. The postoperative pathology confirmed a diagnosis of well-differentiated liposarcoma (Figure 3).

**Figure 3:**
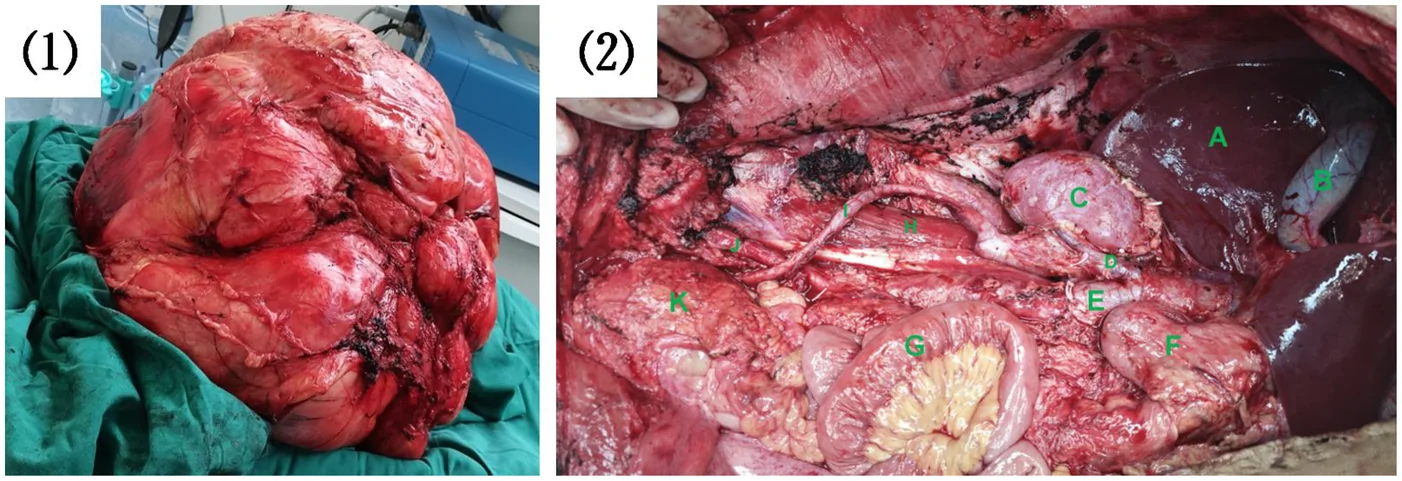
The overall view of postoperative pathology and the remaining organs **(1)** huge tumor after resection; **(2)** normal anatomical map of abdominal organs after tumor resection. A, liver; B, gallbladder; C, right kidney; D, right renal vein; E, inferior vena cava; F, duodenum; G, small intestine; H, psoas major muscle; I, right ureter; J, right iliac artery; K, bladder.

## Discussion

The 2020 World Health Organization (WHO) Classification of Tumors of Soft Tissue and Bone identifies five primary subtypes of liposarcoma: well-differentiated liposarcoma (WDLPS), dedifferentiated liposarcoma (DDLPS), myxoid liposarcoma (MLPS), pleomorphic liposarcoma (PLPS), and myxoid pleomorphic liposarcoma (MPLPS) (4). WDLPS typically manifests as slow-growing masses, commonly found in the retroperitoneum and proximal extremities. Although WDLPS lacks metastatic potential and generally carries an excellent prognosis when completely excised, its behavior varies depending on the location. Tumors originating in the retroperitoneum or mediastinum are prone to local recurrence, contributing significantly to morbidity and mortality. Furthermore, these locations pose a higher risk for progression to dedifferentiated disease (5).The cornerstone of treatment for localized liposarcoma is wide local excision with negative surgical margins. However, the optimal surgical approach for retroperitoneal liposarcoma remains a topic of debate. Some experts advocate for more extensive resections to mitigate the high risk of local recurrence (6).

Previous studies emphasize the perioperative management of giant retroperitoneal liposarcoma. Summary of reported cases and their focuses of retroperitoneal liposarcoma in adults were listed in Table 3.

**Table 3:** Summary of reported cases and their focuses of retroperitoneal liposarcoma .

Reference	Age	Sex	Tumor size	Histopathological	Anaesthesia-related aspects
Luisa M. Recinos/2021 (7)	58	Male	20cm	dedifferentiated liposarcoma	malignant gastric outlet obstruction and acute pancreatitis caused by liposarcoma
Hirotaka Kinoshita/2022 (8)	64	Male	Not mention	liposarcoma	preoperative management of respiratory failure in a patient with a solid giant retroperitoneal tumor with a hemodynamic monitoring system and nasal high-fow therapy (NHFT)
Huaqin Liu/2020 (9)	36	Male	60cm	dedifferentiated liposarcoma	giant intra-abdominal liposarcomas weighing over 20 kg often increase the intra-abdominal pressure (IAP), which has severe effects on the cardiovascular and respiratory systems
Dandan Feng/2015 (10)	66	Male	45cm	liposarcoma	FloTrac/ VigileoTM monitoring system to maintain the stability of circulatory state.
Peidang Fan/2024 (11)	Not applicable	Not applicable	Not applicable	liposarcoma	multidisciplinary team management could result in improved clinical outcomes, higher diagnostic accuracy, and reduced duration of postoperative stays, complications, and perioperative mortality

Preoperative multidisciplinary consultation is critical for managing patients with giant abdominal liposarcomas due to the complexity of their conditions. The implementation of a multidisciplinary team (MDT) approach has significantly improved treatment strategies for high-risk patient during the perioperative period. Most patients underwent surgery after comprehensive risk assessment and, when necessary, optimization of perioperative treatment to reduce potential complications (12).

A previous study highlighted the successful application of a transplant nurse-led MDT model in the postoperative care of patients undergoing simultaneous pancreas and kidney transplantation, which effectively reduced perioperative complications (13). Another study underscored the effectiveness of MDT in clinical and surgical settings, particularly involving the departments of surgery, oncology, and other specialties (11). Specifically, the Department of Anesthesiology has played a crucial role in perioperative care in such surgeries (14, 15), given the multiorgan involvement caused by the tumor.

In our case, following the guidance of MDT, we conducted comprehensive preoperative preparations, including detailed assessment of the potential risk of hemodynamic instability during anaesthesia induction, establishment of large-bore intravenous access and central venous lines, and pre-induction arterial cannulation for continuous blood pressure monitoring and early detection of circulatory changes.

Considering the physiological consequences of inferior vena cava compression and impaired venous return caused by the giant retroperitoneal mass, reverse Trendelenburg positioning and manual tumor displacement during induction were implemented to optimize venous return and reduce the risk of induction-related hypotension. During surgery, adequate blood products were prepared in anticipation of massive blood loss, and a restrictive fluid strategy combined with goal-directed hemodynamic optimization was adopted to avoid cardiac overload while maintaining sufficient organ perfusion. Dopamine and norepinephrine were administered as needed to maintain hemodynamic stability during prolonged tumor resection. Furthermore, anesthetic management was continuously adjusted to accommodate the dynamic physiological transition from tumor compression to tumor decompression after resection, with particular attention to changes in venous return, hemodynamics, respiratory mechanics, and intra-abdominal pressure, as well as the prevention of postoperative pulmonary complications through appropriate intraoperative management and postoperative intensive care.

Comprehensive perioperative management included active temperature maintenance using warming blankets, a forced-air warming system, and warmed intravenous fluids to prevent hypothermia, together with frequent arterial blood gas analyses to optimize electrolyte balance and physiological homeostasis. These measures contributed to an uneventful perioperative course and further highlight the importance of anesthesiologists serving as central members of the multidisciplinary perioperative care team, as has been advocated in the field of perioperative brain health (16).

Several strengths of this case should be noted. First, the tumor was exceptionally large (31 kg; 52 × 38 × 30 cm) and recurrent, with extensive compression and adhesion to major abdominal structures. Second, perioperative planning was conducted through a multidisciplinary team involving anesthesiology, retroperitoneal oncology surgery, urology, gastrointestinal surgery, vascular surgery, and intensive care. Third, invasive arterial monitoring, central venous access, preoperative blood-product preparation, active temperature management, and frequent blood-gas assessment were incorporated into the perioperative strategy.

However, this case also has limitations. First, as a single case report, the observations cannot be generalized to all patients with giant retroperitoneal tumors. Second, intraoperative point-of-care coagulation monitoring, such as TEG or ROTEM, was not performed in this case. Conventional coagulation tests were available preoperatively and postoperatively, but were not repeated intraoperatively. Therefore, real-time assessment of coagulation dynamics was limited.

## Conclusion

In the perioperative management of giant retroperitoneal liposarcoma, anesthesiologists should play a central role in multidisciplinary care by anticipating perioperative risks and adapting anesthetic management to the transition from tumor compression to tumor decompression.

## Data Availability

The raw data supporting the conclusions of this article will be made available by the authors, without undue reservation.
